# Complement C3a/C3aR and C5a/C5aR deposits accelerate the progression of advanced IgA nephropathy to end-stage renal disease

**DOI:** 10.1007/s10238-024-01410-3

**Published:** 2024-06-29

**Authors:** Ying Wang, Shunlai Shang, Shimin Jiang, Guming Zou, Hongmei Gao, Wenge Li

**Affiliations:** Department of Nephrology, China–Japan Friendship Hospital, No. 2 East Yinghuayuan Street, Chaoyang District, Beijing, 100029 China

**Keywords:** IgA nephropathy, Complement C3a/C3aR, Complement C5a/C5aR, Prognosis

## Abstract

IgA nephropathy (IgAN) is still one of the leading causes of end-stage kidney disease (ESRD), and complement system activation is a key to the pathogenesis of IgAN. The role of complement C3a/C3aR and C5a/C5aR in late stage of IgAN remains unknown. Renal specimens of 75 IgAN patients at the stage 4 CKD were stained using immunofluorescence and immunohistochemistry. The primary outcome was a composite of end-stage renal disease (ESRD) and death. Associations of complement components with baseline clinicopathological characteristics and outcomes were assessed using multivariable Cox regression and Spearman analyses. During a median follow-up of 15.0 months, 27 patients progressed to ESRD and none died. Lower eGFR [hazards ratio (HR), 0.827, 95% confidence interval (CI), 0.732–0.935; *P* = 0.002] and glomerular C3 deposition (HR, 3.179, 95% CI, 1.079–9.363; *P* = 0.036) were predictive of time to ESRD in stage 4 CKD IgAN. Higher expression of C3a (*P* = 0.010), C3aR (*P* = 0.005), C5a (*P* = 0.015), and C5aR (*P* < 0.001) was identified in ESRD group than in non-ESRD group. Glomerular C3a/C3aR and C5a/C5aR deposits were both correlated with a lower baseline eGFR, higher baseline 24 h-urinary protein (24 h-UP) and faster decline of eGFR. Besides, C3a and C5a deposits were found in patients with high S (S1) and T (T1/2) scores, respectively. Complement C3a/C3aR and C5a/C5aR in IgAN patients with stage 4 CKD may portend a faster deterioration of kidney function.

## Introduction

IgA nephropathy (IgAN) is still the most prevalent glomerulonephritis worldwide and remains a leading etiological factor of end-stage kidney disease (ESRD) caused by primary glomerulonephritis [1]. The heterogeneous risk of a progressive decline in kidney function decline has posed a challenge for clinicians in identifying these patients over time. Consequently, the effective treatments and accurate biomarkers of disease severity and progression is limited. Typically, IgAN is asymptomatic at an early stage and when diagnosed, some patients have already progressed to severe renal insufficiency. Patients with advanced (stage 4) chronic kidney disease (CKD) are infrequently subjected to renal biopsies for diagnostic purposes and tend to rapidly advance to ESRD, resulting in a limited understanding of the clinicopathological characteristics of this patient population.

Currently, the widely accepted framework for understanding the pathogenesis of IgAN is the “multi-hit hypothesis” [2], with complement (C) activation as one of these pivotal pathogenic steps. The complement system is a key component of the innate immune system [3] and can be initiated through the classical (CP), alternative (AP) and lectin (LP) pathways. A growing body of evidence suggests that complement system activation may associated with glomerular inflammation and renal injury. It is reported that the serum and urinary levels of complement components may be associated with disease severity and prognosis [4, 5]. AP and LP are proved to be activated in 75–90% and 17–25% IgAN patients [6–8], while C1q, a marker of CP, is rare in IgAN [9]. Activation of complement system results in cleavage of C3 and C5, together with the initiation of terminal pathway. C3a and C5a, small cleavage fragments generated by complement activation, are key mediators of inflammation called “anaphylatoxins”. By signaling through their specific receptors, C3a receptor (C3aR) and C5a receptor (C5aR), C3a and C5a can induce diverse biological functions. Liu et al. [10] have reported a correlation between the expression of C3aR and C5aR with the severity of IgAN, however, it remains unclear about their roles in advanced (stage 4 CKD) IgAN patients.

Recognizing the significance of the complement system in IgAN, we conducted this study to clarify C3a/C3aR, C5a/C5aR and their prognostic relevance in IgAN patients with stage 4 CKD.

## Methods

### Study design and participants

**Fig. 1:**
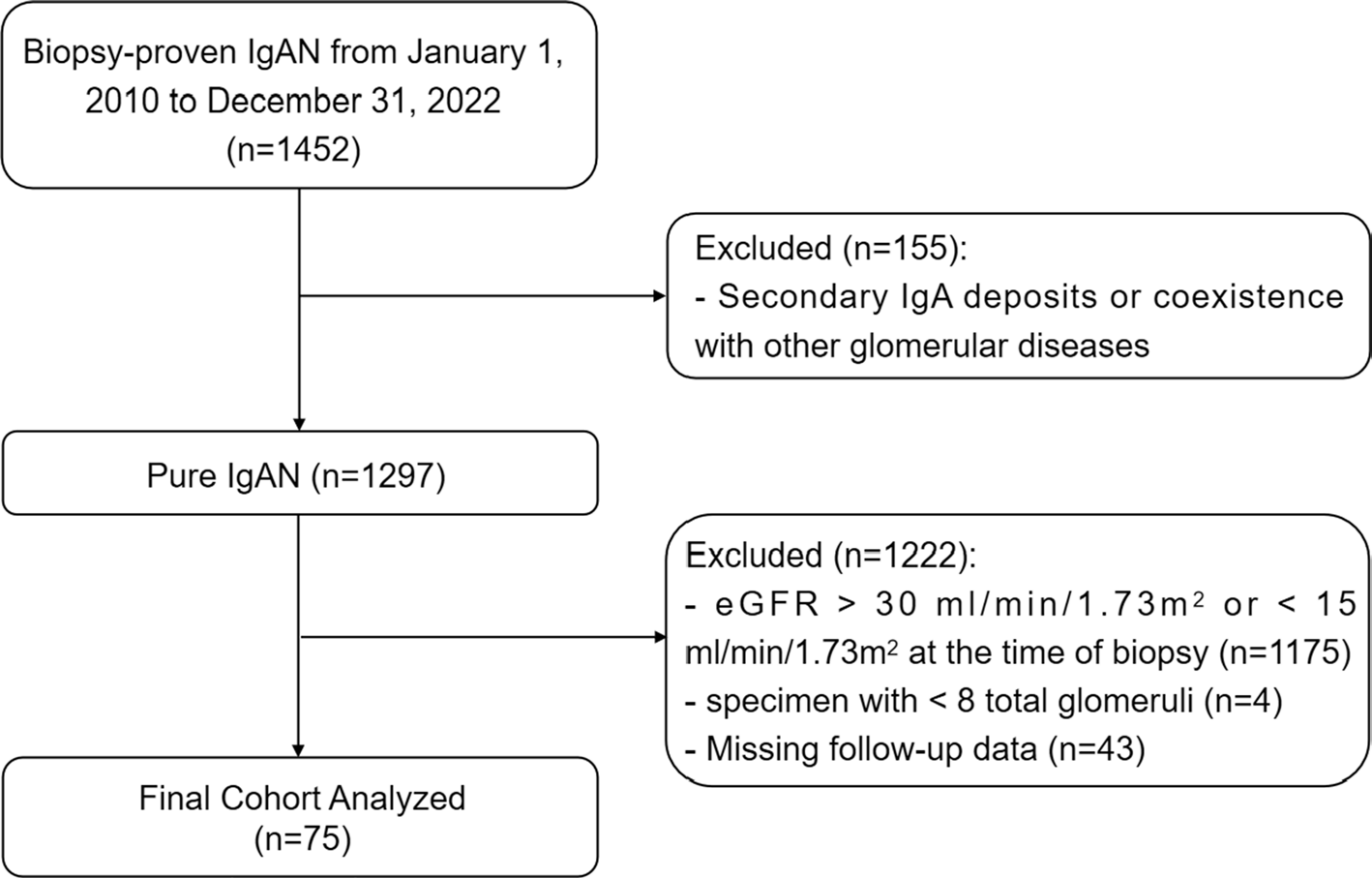
Flowchart of study participants. IgAN, IgA nephropathy; eGFR, estimated glomerular filtration rate; CKD, chronic kidney disease

This was a retrospective study. We included patients with IgAN as the only glomerular disease diagnosis who underwent a renal biopsy at the China-Japan Friendship Hospital between January 1, 2010 and December 31, 2022. Renal biopsies were performed by experienced clinicians in our department. The inclusion criteria were as follows: (1) biopsy-proven IgAN patients with stage 4 CKD at the time of biopsy and (2) age ranging from 18 to 75 years. Primary exclusion criteria included patients (1) with secondary IgA deposits such as severe infection, purpuric nephritis, lupus nephritis, or coexistence of other glomerular diseases; (2) with a biopsy specimen with < 8 total glomeruli; and (3) those without follow-up data (Fig. 1).

This study was approved by the Ethics Committee of the China-Japan Friendship Hospital (2021-113-K71). Since it was a retrospective observational study based on de-identified data, informed patient consent was not deemed necessary. All procedures involving human participants were performed in accordance with the principles of the Declaration of Helsinki.

### Parameters and definitions

Various clinical parameters were obtained from the electronic medical system, including gender, age, body mass index (BMI), mean arterial pressure (MAP), 24 h urinary protein excretion (24 h-UP), urinary red blood cell count (URBC, the highest count before renal biopsy), hemoglobin (Hb), total cholesterol (TC), triglyceride (TG), parathyroid hormone (PTH), uric acid (UA) and serum IgA to C3 ratio. The estimated glomerular filtration rate (eGFR) was calculated using the creatinine-based Chronic Kidney Disease Epidemiology Collaboration (CKD-EPI) equation [11]. We also conducted a retrospective review of post-biopsy usage of renin-angiotensin system (RAS) blockade or immunosuppressive (IS) therapy.

### Endpoints and follow-up

The primary endpoint was defined as a composite of ESRD and death. ESRD was defined as eGFR < 15 ml/min/1.73 m^2^ or the initiation of maintenance dialysis or renal transplantation. The ESRD group was defined as those with the occurrence of the endpoint events, whereas the other group was defined as the non-ESRD group. A 3 year follow-up was conducted. The progression rate of kidney disease was assessed in terms of the mean change in eGFR over the number of follow-up months [ΔeGFR/M = (final eGFR − baseline eGFR)/follow-up time in months].

### Renal biopsy and pathological classification

All the renal biopsy specimens were preserved at the China–Japan Friendship Hospital. Light and electron microscopy, along with immunofluorescence were routinely performed on each biopsy specimen. Renal biopsy specimens were reviewed by investigators blinded to the clinical outcomes.The intensity of direct immunofluorescence staining of C3 deposits in kidney tissue was graded using a semiquantitative method on a scale of 0–4 + (–, no fluorescence at either low or high magnification; ±/trace, no fluorescence at low magnification but somewhat visible at high magnification; +, somewhat visible at low magnification but clearly visible at high magnification; ++, clearly visible at either low or high magnification; +++, clearly visible at low magnification but glaring at high magnification; and ++++, glaring at low magnification and even more glaring at high magnification) [12].

All patients were performed the standardized MEST-C (mesangial [M] and endocapillary [E] hypercellularity, segmental sclerosis [S], interstitial fibrosis/tubular atrophy [T], and crescents [C]) scores according to the revised Oxford Classification [13]. All the specimens were scored by the same renal pathologist who were blinded to the study.

### Immunohistochemistry (IHC) of C3a/C3aR and C5a/C5aR

Paraffin-embedded tissues were sectioned at 3 μm thickness. After dewaxing and hydration treatment, heat-mediated antigen retrieval was performed for C3a and C5a. To retrieve the C3aR and C5aR antigen, pepsin (Zhongshan Golden Bridge Biotechnology, Beijing, China, ZLI-9013) was used at 37 °C for 30 min. The sections were blocked with 3% peroxide-methanol at room temperature for endogenous peroxidase ablation and then incubated with goat serum at room temperature for 30 min. The antibodies used in this study included anti-C3a antibody (Abcam, ab36385, 1:200), anti-C3aR antibody (Abcam, ab140788, 1:200), anti-C5a antibody (Abcam, ab281923, 1:200), and anti-C5aR antibody (Abcam, ab252435, 1:1000). After immunostaining, all sections were counterstained with hematoxylin. Immunohistochemical images were obtained using a Moticam 2506 instrument (Motic, Fujian, China) from sections observed microscopically at × 400 magnification (Nikon, Tokyo, Japan). Images were obtained using an integrated digital camera system (Nikon). Immunoreactivity was evaluated semi-quantitatively in a blinded manner. Image Pro-Plus (IPP) computer image analysis software (Media Cybernetics, Bethesda, MD, USA) was used to analyze the pixel density of the stained areas, and to quantify the protein levels.

## Statistical analysis

Continuous data were considered nonparametric and were presented as medians and interquartile ranges (IQR). Differences were analyzed using the Mann–Whitney U non-parametric test. Categorical variables were reported as percentages and analyzed using the chi-square or Fisher’s exact test. Univariate and multivariate Cox proportional hazard models were used to calculate the hazard ratios (HRs) and 95% confidence intervals (CIs) for variables related to the composite outcomes. Spearman’s correlation analysis was performed to assess the relationship between baseline eGFR, 24 h-UP, and the mean change in renal function with the expression of complement components. Statistical analysis was performed using SPSS software (version 24.0; IBM Corp, Armonk, NY, USA) and GraphPad Prism 8.0. Two-sided *P* values less than 0.05 were considered significant.

## Results

### Baseline and pathological characteristics

In our study, we included 75 IgAN patients with stage 4 CKD at the time of renal biopsy aged 18–75 years, who underwent renal biopsy at the China-Japan Friendship Hospital between 2010 and 2022. The clinical characteristics of the patients are summarized in Table 1. The median age of all patients was 42.0 (IQR 31.0–50.0) years, and 38 (50.7%) patients were male. The median eGFR was 24.9 (20.7–26.4) ml/min/1.73 m^2^. The median 24 h-UP was 2.8 (1.6–4.3) g/d. By the end of the follow-up period, 27 (36.0%) of the 75 patients had progressed to ESRD. At baseline, there were no significant differences in key biochemical parameters such as Hb (117.0 vs. 110.0 g/L, *P* = 0.33), eGFR (25.1 vs. 22.0 ml/min/1.73 m^2^, *P* = 0.14) and 24 h-UP (2.5 vs. 3.5 g/d, *P* = 0.07) between the ESRD group and non-ESRD group. Direct immunofluorescence microscopy for C3 staining showed completely negative, 1+ ~ 2+, and > 2+ staining in 4 (5.3%), 28 (37.3%), and 43 (57.3%) patients, respectively. As a feature of IgAN, C3 deposition was observed predominantly in the mesangium. It should be noted that there were really significant differences in the numbers of MAP and 24 h-UP, but there were no statistical significance in these two parameters between non-ESRD and ESRD groups, perhaps because of the small sample size and methodological limitations. As shown in Table 1, patients with a strong intensity of glomerular C3 deposition and a lower baseline serum C3 were at a high risk of renal progression (*P* = 0.012 and 0.001, respectively).

**Table 1:** Baseline information of IgAN patients with stage 4 CKD

Parameters	Overall (n = 75)	Non-ESRD (n = 48)	ESRD (n = 27)	*P*
*Clinical findings*				
Age (y)	42.0 (31.0–50.0)	43.0 (31.2–57.8)	39.0 (30.0–46.0)	0.06
Gender, M (%)	38 (50.7)	24 (50.0)	14 (51.9)	0.93
SBP (mmHg)	137.0 (126.0–154.0)	136.5 (125.3–156.5)	139.0 (127.0–154.0)	0.71
DBP (mmHg)	90.0 (78.0–98.0)	87.5 (75.5–95.8)	93.0 (82.0–101.0)	0.07
MAP (mmHg)	102.7 (96.7–116.7)	100.8 (96.2–112.7)	110.3 (97.7–118.0)	0.20
Hypertension, n (%)	48 (64.0)	32 (66.7)	16 (59.3)	0.41
Use of RASi, n (%)	29 (38.7)	21 (43.8)	8 (29.6)	0.31
Use of IS, n (%)	38 (50.7)	26 (54.2)	12 (44.4)	0.29
*Laboratory findings*				
Hb (g/L)	112.0 (104.0–123.0)	117.0 (103.0–124.8)	110.0 (104.0–122.0)	0.33
SCr (μmol/L)	248.8 (207.7–280.0)	238.2 (201.2–277.8)	258.0 (212.0–294.5)	0.10
eGFR (ml/min/1.73 m^2^)	24.9 (20.7–26.4)	25.1 (22.2–26.9)	22.0 (19.9–26.1)	0.14
UA (μmol/L)	469.0 (422.0–515.0)	471.5 (422.3–525.0)	459.0 (397.0–497.0)	0.68
PTH (pg/mL)	75.2 (45.0–132.7)	70.6 (44.5–131.9)	81.9 (45.0–133.6)	0.24
TC (mmol/L)	5.3 (4.3–6.5)	5.3 (4.3–6.1)	5.3 (4.3–6.9)	0.64
TG (mmol/L)	1.8 (1.6–2.5)	1.8 (1.4–2.6)	1.9 (1.6–2.4)	0.72
24 h-UP (g/d)	2.8 (1.6–4.3)	2.5 (1.4–3.6)	3.5 (2.3–4.7)	0.07
URBC (/HPF)	12.0 (4.8–54.2)	11.5 (3.4–84.3)	14.4 (4.1–41.8)	0.52
sIgA	298.0 (222.0–417.0)	299.0 (232.0–454.8)	288.0 (203.0–378.0)	0.15
sC3	87.6 (76.5–105.0)	97.6 (80.0–112.0)	77.8 (71.8–87.6)	**0.001**
Serum IgA/C3	3.5 (2.6–5.0)	3.6 (2.8–5.0)	3.4 (2.6–4.8)	0.50
*Glomerular C3 deposition, n* (%)				**0.012**
C3 –	4 (5.3)	4 (8.3)	0 (0.0)	
C3 1 + ~ 2+	28 (37.3)	22 (45.8)	6 (22.2)	
C3 > 2+	43 (57.3)	22 (45.8)	21 (77.8)	

SBP, systolic pressure; DBP, diastolic pressure; MAP, mean arterial pressure; RASi, renin-angiotensin system inhibitor; IS, immunosuppressive agent; Hb, hemoglobin; SCr, serum creatinine; eGFR, estimated glomerular filtration rate; UA, uric acid; PTH, parathyroid hormone; TC, total cholesterol; TG, triglyceride; 24 h-UP, 24 h urinary protein excretion; URBC, urinary red blood cells; sIgA, serum IgA; sC3, serum C3

### Association of C3 deposition and clinical outcomes

**Fig. 2:**
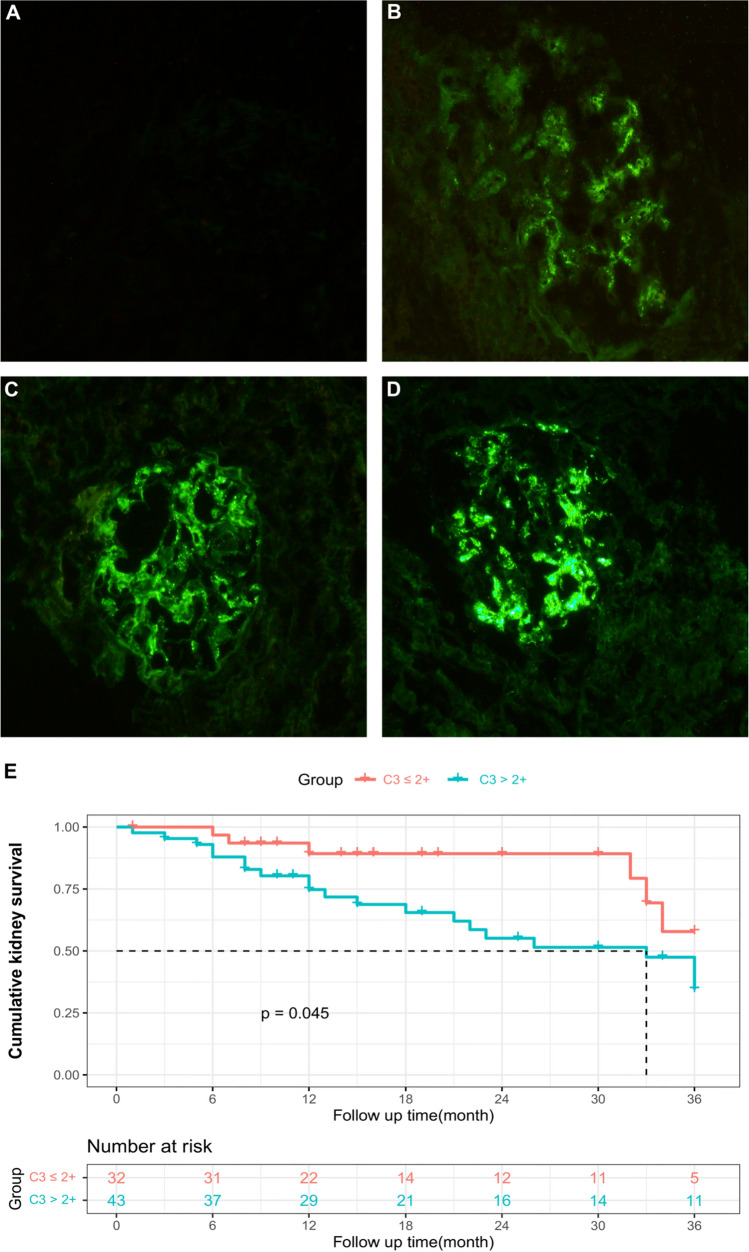
Representative immunofluorescent staining (**A**–**D**) for C3 in IgAN patients and Kaplan–Meier survival curves (**E**) for primary outcomes according to glomerular C3 deposits. **A** No fluorescence observed under both low magnification and high magnification; **B** 1+: faintly visible under low magnification but clearly visible under high magnification; **C** 2+: can be clearly visible under low or high magnification lenses; **D** 3+: Clearly visible under low magnification, more dazzling under high magnification; **E** The fluorescence intensity of C3 deposition and the K–M survival curve of kidney survival. Note: As there were only 4 patients in C3– group, we combined this group with C3 1+~2+ group

During a median follow-up period of 15.0 months, 27 (36.0%) of 75 patients with stage 4 CKD reached the primary endpoint. The median renal survival time in the ESRD group was 13.0 (7.0–32.0) months, while the length of the follow-up time in the non-ESRD group was 15.5 (10.0–36.0) months. No patients died during the 3-year follow-up. Patients with a high immunofluorescence grade were prone to progress into ESRD, 6 (22.2%) and 21 (77.8%) patients with 1 +  ~ 2 + and > 2 + C3 stain, respectively. Figures 2A-D illustrate the degrees of C3 (– ~ 3 +) staining in the glomeruli. Figures 2E shows the Kaplan–Meier survival curves for the composite outcome stratified by glomerular C3 deposition. It suggested > 2 + glomerular C3 deposition was associated with a higher risk of an adverse kidney outcome compared to those with ≤ 2 + glomerular C3 deposition (*P* = 0.045). Multivariate Cox analysis also indicated glomerular C3 deposits [hazards ratio (HR), 3.179, 95% confidence interval (CI), 1.079–9.363; *P* = 0.036] and baseline eGFR level [HR, 0.827, 95% confidence interval (CI), 0.732–0.935; *P* = 0.002] as two independent risk factors for renal progression in IgAN. However, baseline 24 h-UP showed no predictive significance in our Cox analyses. This may be because of the limited sample size, methodological limitations and the different conditions of patients after hospitalization from outside, which might affect the proteinuria results. The results of the univariate and multivariate Cox proportional hazards analyses are shown in Table 2.

**Table 2:** Univariate and multivariate Cox regression analysis between candidate predictors and survival of IgAN patients with stage 4 CKD

Variables	Univariate analysis		Multivariate analysis	
	HR (95%CI)	*P*	HR (95%CI)	*P*
Gender	0.800 (0.370–1.371)	0.57		
Age	0.967 (0.935–1.000)	0.047	0.965 (0.929–1.004)	0.075
MAP	1.019 (0.997–1.041)	0.08		
Hb	0.988 (0.965–1.012)	0.32		
eGFR	0.839 (0.748–0.940)	0.003	0.827 (0.732–0.935)	**0.002**
UA	0.999 (0.995–1.004)	0.71		
PTH	1.000 (0.998–1.002)	0.92		
TC	1.132 (0.908–1.411)	0.27		
TG	1.123 (0.806–1.566)	0.49		
URBC	0.999 (0.995–1.002)	0.42		
24 h-UP	1.115 (0.941–1.321)	0.21		
sIgA	0.997 (0.994–1.001)	0.15		
sC3	0.971 (0.951–0.992)	0.007	0.978 (0.955–1.001)	0.057
IgA/C3	1.004 (0.973–1.272)	0.97		
C3 deposition	3.286 (1.199–9.007)	0.02	3.179 (1.079–9.363)	**0.036**
Hypertension	0.839 (0.385–1.831)	0.66		
RASi	0.456 (0.196–1.061)	0.07		
IS	0.767 (0.352–1.671)	0.51		

MAP, mean arterial pressure; RASi, renin-angiotensin system inhibitor; IS, immunosuppressive agent; Hb, hemoglobin; eGFR, estimated glomerular filtration rate; UA, uric acid; PTH, parathyroid hormone; TC, total cholesterol; TG, triglyceride; 24 h-UP, 24 h urinary protein excretion; URBC, urinary red blood cells; sIgA, serum IgA; sC3, serum C3

### Expression of glomerular C3a, C5a and their receptors in advanced IgAN

**Fig. 3:**
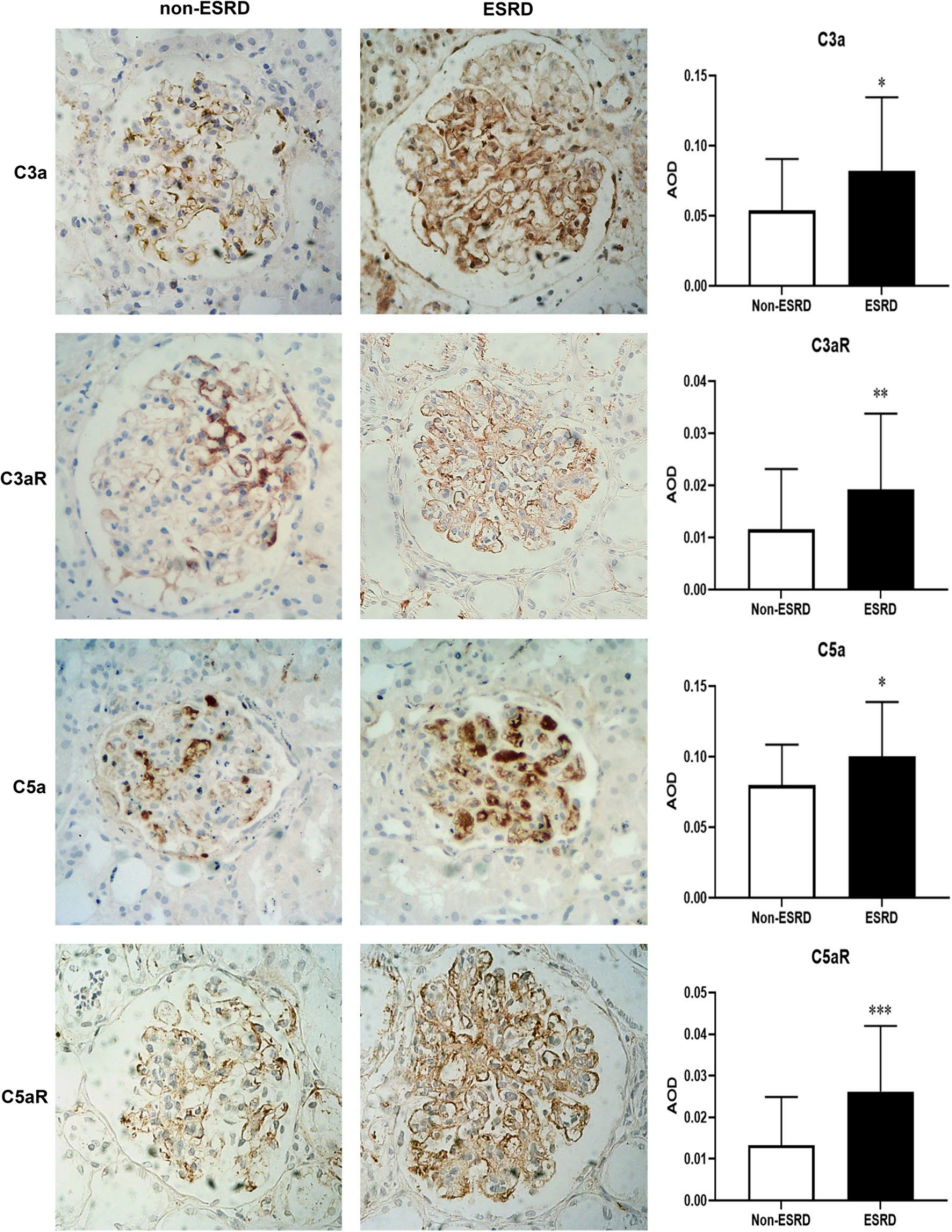
Immunohistochemical staining suggests increased expression of C3a, C3aR, C5a, C5aR in advanced IgAN (× 400), with significant differences between ESRD and non-ESRD groups. AOD, average optical density. *P < 0.05, **P < 0.01, ***P < 0.001

As previously mentioned, the complement system plays an important role in IgAN pathogenesis, and our IHC results provide supporting evidence for this hypothesis. Strong C3a and C5a deposits were found mainly in mesangium in advanced IgAN patients. Meanwhile, notably higher expressions of C3a and C5a were observed in the ESRD group than in the non-ESRD group (*P* = 0.010 and 0.015, respectively). Consequently, the expression levels of C3aR and C5aR also showed correspondingly increases in patients with stage 4 CKD, especially in the ESRD group (*P* = 0.005 and < 0.001, compared to the non-ESRD group, respectively). The IHC staining results are shown in Fig. 3. According to our findings, in IgAN patients with stage 4 CKD, increased expressions of C3aR and C5aR might be predictive of a poor prognosis.

### Correlation of glomerular C3a/C3aR and C5a/C5aR deposits with renal function and pathological features

**Fig. 4:**
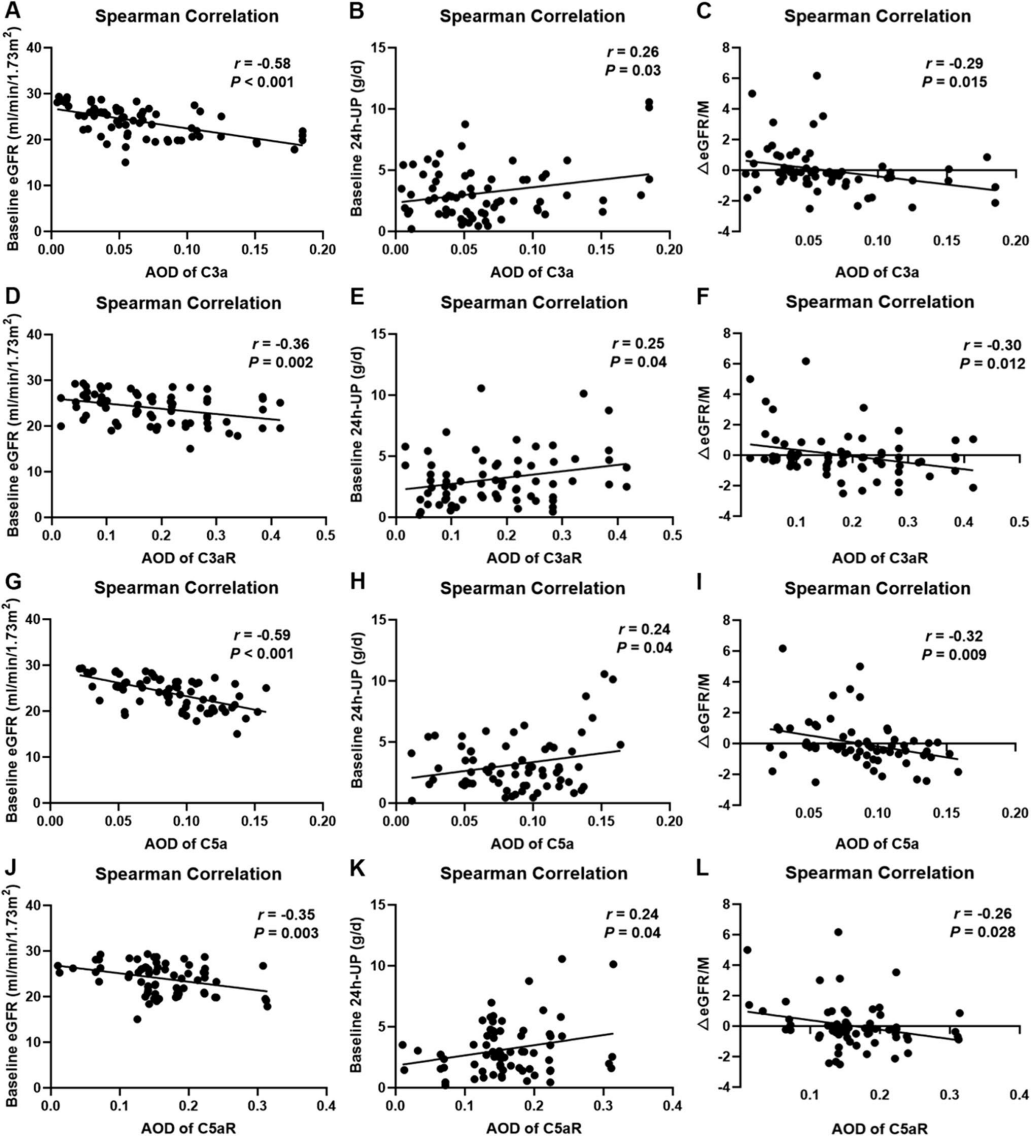
Spearman correlation analysis between C3a/C3aR, C5a/C5aR and baseline eGFR, baseline 24 h-UP, ΔeGFR/M. The results suggested that C3a (**A**, *r* = − 0.58, *P* < 0.001), C3aR (**D**, *r* = − 0.36, *P* = 0.002), C5a (**G**, *r* = − 0.59, *P* < 0.001) and C5aR (**J**, *r* = − 0.35, *P* = 0.003) were all negatively correlated with baseline eGFR, while positively with baseline 24 h-UP (**B**, **E**, **H**, **K**). Deposits of C3a/C3aR and C5a/C5aR could accelerate the mean rate of renal function decline (**C**, **F**, **I**, **L**)

To further investigate the correlation between complement activation with renal function and pathological features, the mean change in eGFR during the follow-up period (ΔeGFR/M) was calculated according to glomerular C3 deposition. Though there was no significant difference in the baseline eGFR between the groups stratified by C3 deposition, patients with > 2+ glomerular C3 deposition were prone to reach a lower median eGFR during the 3 year follow-up (15.2 ml/min/1.73 m^2^ vs. 22.4 ml/min/1.73 m^2^, *P* < 0.001). Meanwhile, patients with > 2+ glomerular C3 deposition showed a faster mean decline rate of eGFR than those with ≤ 2+ glomerular C3 deposition [− 0.3 (− 0.9– − 0.1) vs. − 0.1 (− 0.5–1.2), *P* < 0.001], indicating the important role of complement on the progression of renal function in IgAN patients with stage 4 CKD. The results of glomerular C3 deposition and changes in eGFR were detailed in (Table 3). Spearman’s correlation analysis was conducted to explore the association between C3a and C5a deposits, baseline eGFR, 24 h-UP and ΔeGFR/M. As shown in Fig. 4A, D, glomerular C3a deposition (*r* = − 0.58, *P* < 0.001) and high C3aR expression (*r* = − 0.36, *P* = 0.002) were negatively correlated with baseline eGFR, but positively with baseline 24 h-UP (Fig. 4B, E, *P *= 0.03 and 0.04, respectively). Similarly, C5a deposition and high C5aR level were correlated with lower baseline eGFR and increased baseline 24 h-UP (Fig. 4G, H, J, K). Meanwhile, patients with significant C3a/C3aR and C5a/C5aR expressions were more likely to show a faster decline of renal function. Our findings suggested that local C3a/C3aR and C5a/C5aR deposits in kidneys might accelerate the deterioration of renal function in IgAN patients of stage 4 CKD.

**Table 3:** Mean change rate of eGFR in IgAN patients with stage 4 CKD stratified by C3 deposition

eGFR (ml/min/1.73 m^2^)	Glomerular C3 deposition		
	≤ 2+	> 2+	*P* value
Baseline eGFR	25.3 (22.2–27.4)	23.6 (20.0–26.4)	0.14
Final eGFR	22.4 (15.6–40.0)	15.2 (11.4–26.9)	** < 0.001**
△eGFR/M	− 0.1 (− 0.5–1.2)	− 0.3 (− 0.9–− 0.1)	** < 0.001**

Variables in the table are presented in the form of median (interquartile range). eGFR, estimated glomerular filtration rate; ΔeGFR/M, mean change rate of eGFR during the follow-up (months)

**Fig. 5:**
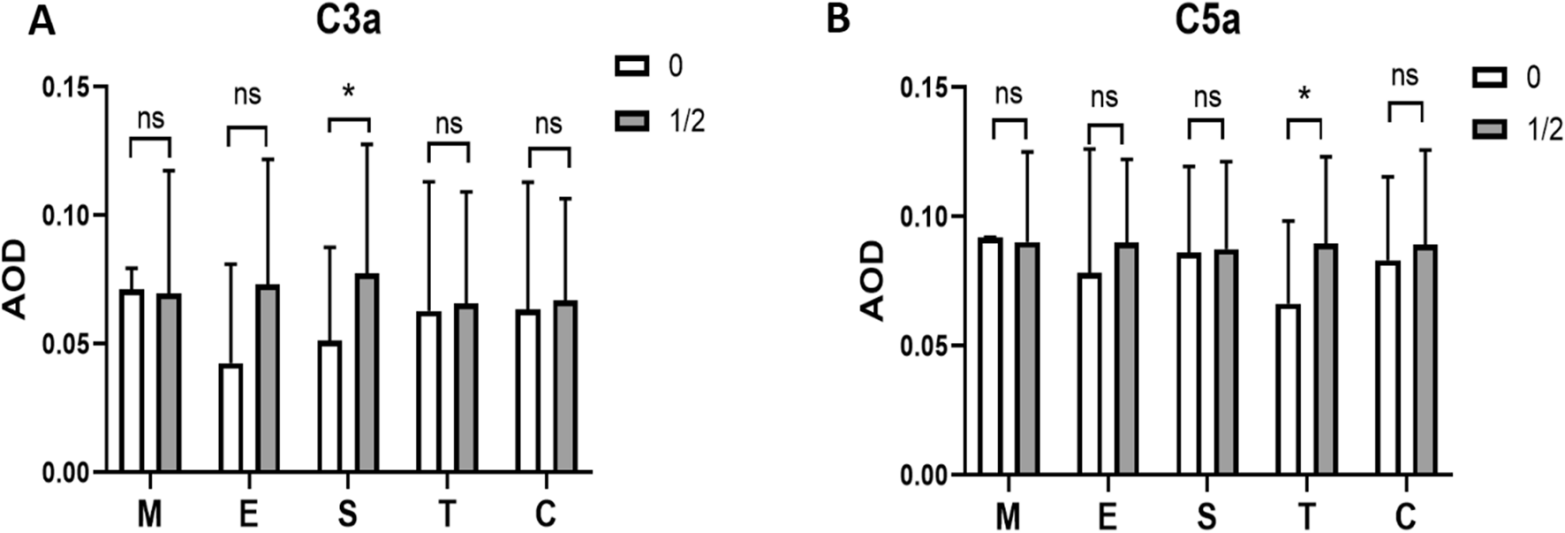
The correlation between glomerular C3a or C5a deposit and MEST-C scores. Patients with S1 showed a higher AOD of C3a (**A**), while those with T1/2 showed a higher AOD of C5a (**B**)

The Oxford classification is acknowledged as an important pathological index of IgAN. We thus also conducted the correlation between complement components and MEST-C scores. While no significant correlations were found between the C3 immunofluorescence intensity and the Oxford classification (Table 4), glomerular C3a and C5a deposits showed some connections with S score and T score, respectively (Fig. 5).

**Table 4:** Pathologic features of advanced IgAN patients stratified by C3 immunofluorescence intensity

The Oxford classification	Glomerular C3 deposition		
	≤ 2+	> 2+	*P* value
M1	30 (93.8%)	43 (100.0%)	0.18
E1	28 (87.5%)	41 (95.3%)	0.39
S1	18 (56.3%)	28 (65.1%)	0.44
T1/2	28 (87.5%)	37 (86.0%)	0.84
C1/2	15 (46.9%)	23 (53.5%)	0.57

## Discussion

IgAN patients may have already experienced various degrees of renal dysfunction at the time of diagnosis due to its insidious nature at the early stage. Patients with stage 4 CKD are seldom biopsied for a definite diagnosis because of the risk of complications such as bleeding, resulting in a limited clinical and pathological data for this group of patients. The activation of the complement system is one of the key factors involved in IgAN renal injury. Our study is the first to explore the correlation between the expression of complement C3a/C3aR, C5a/C5aR and prognosis in IgAN patients with severe renal dysfunction. The results indicate that in patients with late stage IgAN (stage 4 CKD during renal biopsy), complement terminal pathway was significant activated in kidneys, and local deposits of complement C3a and C5a, as well as high expression of C3aR and C5aR were closely related to baseline renal function and proteinuria levels, and might accelerate the progression of renal function to ESRD.

The complement system is not only an important effector of the innate immune system, but an important regulatory factor of the acquired immune system. Activation of the complement system is observed in many glomerulonephritis, such as anti-neutrophil cytoplasmic antibodies (ANCA) associated vasculitis (AAV) [14]. Kidneys are particularly susceptible to complement mediated disease attacks. Up to now, it is generally recognized that complement activation plays a role in many kidney diseases, such as atypical hemolytic uremic syndrome (aHUS), C3 glomerulopathy (C3G), and even membranous nephropathy (MN) [15, 16]. Activation of two or more complement pathways can be detected in almost all kinds of autoimmune glomerulonephritis. Recently, a growing body of evidence has highlighted the role of complement activation in IgAN. Studies have shown that the fluorescence intensity of C3 deposition in the mesangial region is related to the severity and progression of IgAN. Our research results also showed that in the late stage (stage 4 CKD) of IgAN, patients with glomerular C3 deposition were more likely to progress into ESRD. The K–M survival curve further indicated that those with high fluorescence intensity of C3 (> 2 +) in renal tissues might result in a significantly shorter kidney survival time compared to those with lower intensity, indicating that degree of complement activation has a certain predictive effect on the prognosis of the disease.

At present, various studies have shown that there are at least two complement pathways activated in IgAN, mainly through the activation of alternative, lectin, and terminal pathways. Chiu et al. [17] found that factor B expression can be observed in the renal tissues of IgAN patients, but not in patients with focal segmental glomerulosclerosis (FSGS). In addition, the serum level of fragment Ba is positively correlated with the clinical activity of the disease (including urinary protein/creatinine ratio and renal function), as well as with the pathogenic galactose deficient IgA1 (Gd IgA1) antibody. Abnormal deposition of complement regulatory factors is also associated with IgAN, such as the deposition of factor H-related protein 1 (CFHR1) and 5 (CFHR5), which are more easily detected in patients with more severe conditions [4]. Activation of complement classical and lectin pathways can both produce C4d, but C4d expression can still be observed in patients with C1q negative IgAN. Therefore, C4d is believed to mainly originate from lectin pathway. Clinical observational studies have found that MBL and C4d deposits in glomeruli, even in renal arteriolar vessel walls, are closely correlated to the severity and poor prognosis of IgAN [18–24]. Unlike other glomerular diseases, it is currently believed that C1q does not play a crucial role in IgAN. Some previous studies have suggested that there is no significant correlation between mesangial C1q deposition and clinical characteristics of IgAN patients. However, others have proposed that C1q deposition was associated with poor renal prognosis and severe renal pathological changes [25, 26]. Another multicenter, prospective study involving 1071 IgAN patients showed that C1q deposition was a predictive factor for poor prognosis during an average follow-up period of 41.89 months [27].

The activation of any pathway of complement can lead to the generation of C3 converting enzyme, which cleaves C3 to form C3a and C3b, and formation of membrane attack complexes (MAC), namely C5b-9. This process ultimately produces C3a and C5a. C3a and C5a are currently considered to be two important anaphylatoxins that can mediate acute inflammatory responses after complement activation. The combination of C3a/C3aR and C5a/C5aR has various biological effects, which can regulate the proliferation and function of various cells in the human body, such as T cells, eosinophils/basophils, mast cells, etc. It also plays an important role in various inflammatory diseases and even tumor progression and metastasis [28, 29]. The correlations between C3a/C3aR and C5a/C5aR with various kidney diseases, even renal failure after kidney transplantation, have also been increasingly valued. For example, Li L et al. found that antagonists of C3aR and C5aR alleviate endothelial myofibroblast transformation through Wnt/β-Catenin signaling pathway in diabetes nephropathy [30].

C3a/C3aR is reported to be associated with disease severity in Alzheimer’s disease, asthma, and even coronary artery disease [31–33]. Under normal circumstances, C3aR is highly expressed in renal tubular epithelial cells, while only a small amount is expressed in podocytes and glomeruli [34]. Currently, there is some controversy over the role of C3a/C3aR in the occurrence of kidney diseases and tissue damage. Mizuno M et al. found that C3aR is highly expressed in the glomeruli of patients with lupus nephritis, especially in active lesions of type IV lupus nephritis [35]. It has been reported that in IgAN, deposits of C3a and C3aR in glomerular mesangial cells might be related to the Haas grading [10]. Our research results indicated that the expression of glomerular C3a and C3aR in IgAN patients with stage 4 CKD was significantly increased, mainly in the mesangial, endothelial and epithelial cells, especially in those with rapid renal function progression (ESRD group). Spearman correlation analysis suggested that the expression intensity of C3a and C3aR was significantly negatively correlated with baseline eGFR and the mean decrease rate of eGFR, indicating that C3a/C3aR may promote the progression of renal function in advanced IgAN patients.

C5a is also an important inflammatory mediator and another marker of terminal pathway activation. Similar to C3a/C3aR, C5a can produce various effects after binding to its specific receptor C5aR. Up to now, studies on C5a and its receptors mainly focus on immune cells and some certain neurological diseases. Renal mesangial cells have been also reported to express C5aR [36]. Experiments have found that inhibiting C3aR and C5aR could alleviate the proliferation of cultured human mesangial cells and reduce proteinuria levels IgAN model mice induced by in Sendai virus [37]. Other studies have shown a certain correlation between serum C5a levels and the crescent score of IgAN Oxford Classification [38]. In IgAN patients with stage 4 CKD, the expression of C5a and C5aR is approximately the same as that of C3a and its receptors in the glomeruli. C5a and C5aR deposits can also be detected in the mesangial, endothelial and epithelial cells. C5a/C5aR expression level was significantly high in patients with stage 4 CKD, especially those progressing to ESRD. Besides, the expression of C5a and C5aR in this group of patients showed a significant negative correlation with the baseline renal function and its decline. Studies have shown that the expression of C3a/C3aR and C5a/C5aR in IgAN renal tissue is significantly correlated with the level of proteinuria in patients [10]. In our study, the expression of C3a, C5a, and their receptors also showed a negative correlation with baseline 24 h proteinuria levels. Our preliminary analysis displayed the possible relations of local C3a and C5a with S and T scores in advanced IgAN patients. However, due to the limited sample size, further validation will be needed in larger studies in the future.

Generally, most mild IgAN patients do not require immunosuppressive therapy, for traditional treatments such as ACEI/ARB essentially meet the requirements of disease control [39]. In the past decade, a series of studies have been conducted on the pathogenesis of IgAN, including the role of the complement system, which is involved in many diseases due to its abnormal activation. This has driven the development process of related therapeutic drugs [40], and has also initiated multiple clinical trials to further evaluate the risks and benefits of various complement system inhibitors in IgAN treatment [8]. Several complement inhibitors, such as C3 inhibitor APL-2, C5a receptor antagonist avacopan (also known as CCX168), selective oral B-factor inhibitor LNP023, monoclonal antibody OSM721 against MBL associated serine protease 2 (MASP-2), and inhibitors of C5b-9, have mostly been in the phase 2 or phase 3 clinical trials. Our research findings may suggest that IgAN patients may benefit from complement inhibition, maybe even in the advanced IgAN patients, but further exploration based on clinical evidence will still be needed to clarify their efficacy and safety.

Although this is a study focused on patients with advanced IgAN, there are still some limitations. Firstly, due to the high risk of complications such as bleeding, patients with stage 4 CKD seldom undergo renal biopsy. Therefore, conclusions above still need to be explored and validated through larger scale and multi-center studies. Secondly, retrospective research has methodological limitations itself, and we cannot collect the comprehensive pre-biopsy information of every patients completely. If patients have used immunosuppressants before, this may have a certain impact on the expression of complement. In addition, our study mainly investigated the deposition of complement components in the glomeruli, without concerning the expression of complement in the tubulointerstitium and renal blood vessels, which may also mediate kidney damage. Finally, the relationship between the deposition of complement C3a and C5a, as well as their receptor expression, and the pathological characteristics and scores of IgAN still needs further exploration and verification in the future.
